# Phalloplasty Using a Scrotal Fasciocutaneous Rotation Flap Following a High-Voltage Electrical Burn: A Case Report

**DOI:** 10.7759/cureus.92140

**Published:** 2025-09-12

**Authors:** Joselyn Ugalde Rivera, Edgar Andrés Perea Celestín, Luis Miguel Jiménez Granados, Juan Eduardo Sánchez Núñez, Juan Felipe García Silva

**Affiliations:** 1 Surgery, Hospital General de Querétaro, Querétaro, MEX; 2 Medicine, Universidad Anáhuac Querétaro, Querétaro, MEX; 3 Urology, Centro Médico ABC Observatorio, Ciudad de México, MEX

**Keywords:** electrical burns, genital reconstruction, microsurgery, phalloplasty, scrotal flap

## Abstract

High-voltage electrical burns in the genital area are a rare but serious condition, posing significant challenges for surgical management and functional recovery. These injuries can severely compromise penile anatomy and function, leading to complications such as erectile dysfunction, infections, necrosis, and contractile scarring, which significantly affect the patient's quality of life. Penile reconstruction in these cases is complex and requires specialized microsurgical techniques to achieve both functional and esthetic outcomes. We report the case of a 20-year-old male patient with penile injury secondary to high-voltage electrical trauma, managed with scrotal fasciocutaneous flap reconstruction. The surgical technique used is described, including flap design, incision planning, dissection, and two-layer closure. The results demonstrate satisfactory skin coverage and postoperative functionality, with good visual acceptance and minimal donor site complications. This report aims to highlight the role of scrotal flap phalloplasty in functional penile reconstruction following electrical burns. Phalloplasty with a scrotal flap is a valuable option in these cases, providing anatomical and functional benefits. Multidisciplinary care is essential to optimize outcomes and reduce long-term complications.

## Introduction

Penile reconstruction is a complex but essential surgical intervention in cases of extensive genital tissue loss, including traumatic injuries, severe burns, or congenital and acquired defects. Its primary goal is to restore both the structure and function of the penis [1]. High-voltage electrical burns are among the most severe genital injuries, often causing extensive tissue necrosis that necessitates surgical reconstruction [1].

In burn-related cases, the free radial forearm flap is the most frequently employed technique [1]. Alternatively, an innovative approach involves designing a flap using scrotal skin to cover damaged penile areas, given its high healing potential and favorable compatibility with reconstructive needs. The selection of this technique depends on factors such as the extent of injury, the availability of local tissue, and the goal of restoring both erectile function and the ability to urinate [2]. High-voltage injuries typically cause full-thickness burns (third-degree), with extensive necrosis and potential loss of penile and scrotal structures, necessitating early debridement and reconstruction [3].

A detailed understanding of penile anatomy is crucial when considering surgical options. The penis consists of three cylindrical structures: two corpora cavernosa, responsible for erection, and the corpus spongiosum, which surrounds the urethra [2]. The vascularization of these tissues is intricate, comprising a network of arteries and veins that supply blood during erection. Electrical injuries often compromise both the structure and function of these components, making preservation of erectile and urinary function a key goal during reconstruction.

The physical implications of penile reconstruction are considerable, as the objective extends beyond restoring the anatomical integrity of the organ to reestablishing its function. Postoperative complications may include erectile dysfunction, urethral stricture, sensory loss, or fistula formation, depending on the surgical technique employed [4].

## Case presentation

Accident and emergency department admission

A 20-year-old male patient with a history of substance abuse, no chronic degenerative diseases, and no prior surgical history presented to the emergency department in December 2024, approximately one hour after sustaining trauma. The incident occurred while the patient was smoking on the second floor of a building and accidentally came into contact with a high-voltage electrical cable, resulting in an electric shock and a fall of approximately 2 m. He was later found by paramedics, conscious and oriented, reporting intense generalized pain, predominantly in the genital area.

**Figure 1:**
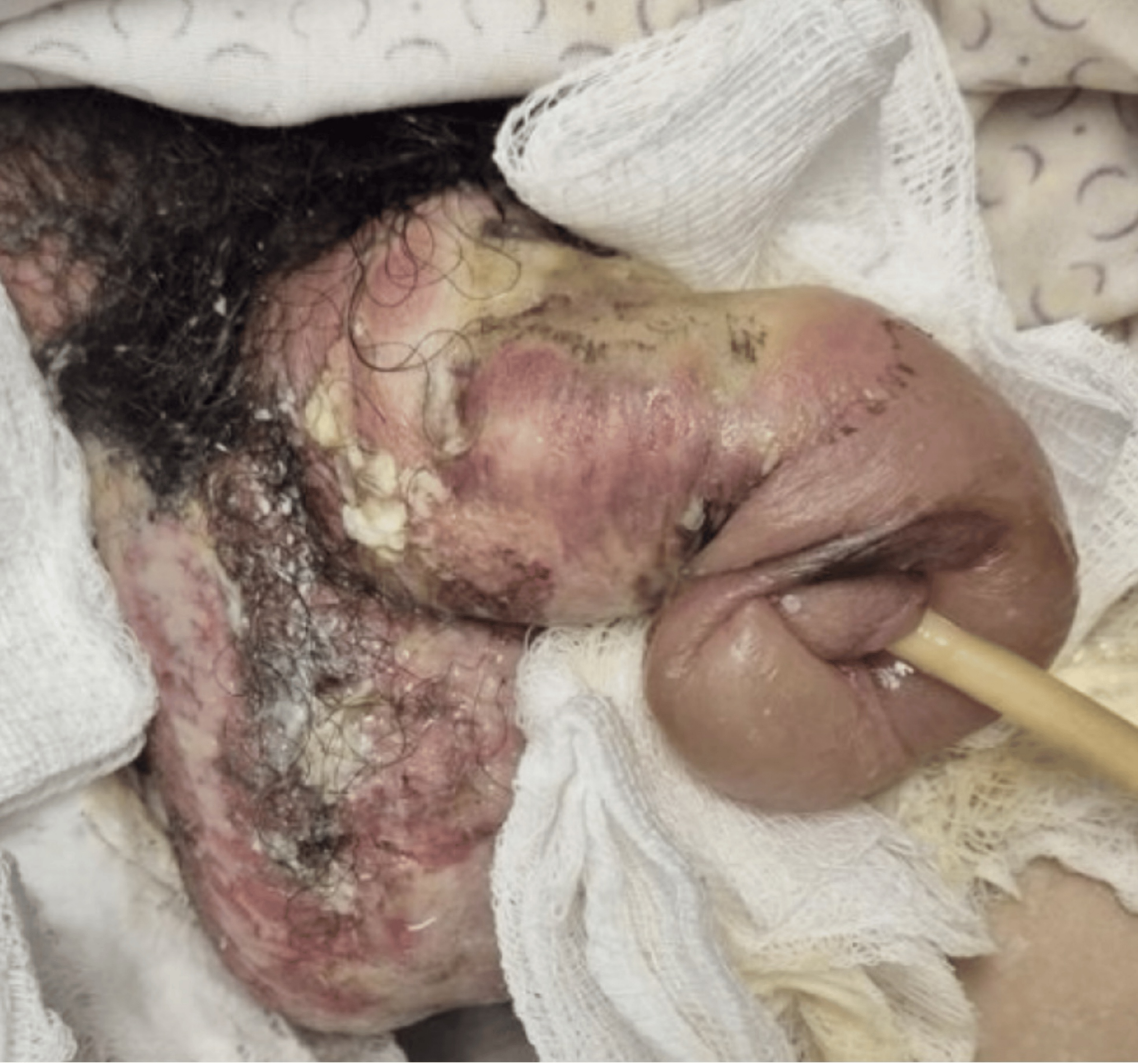
High-voltage electrical burn of the penis and scrotum at emergency department admission Initial clinical image showing extensive thermal damage to the penile and scrotal tissues following accidental contact with a high-voltage electrical cable. Superficial and deep second-degree burns involving the penis and scrotum

**Figure 2:**
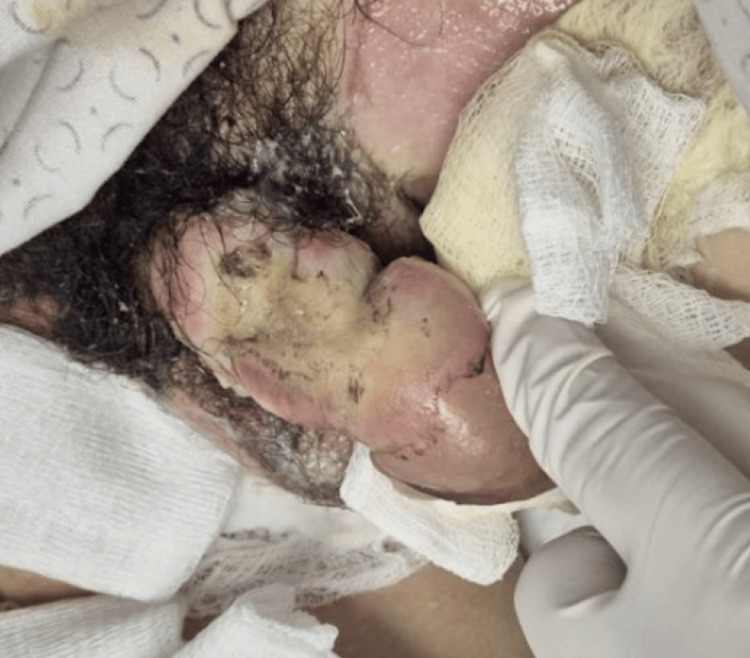
Dorsal view of the injured penis at emergency department admission Clinical image showing superficial and deep second-degree burns on the dorsal surface of the penis. Genital examination revealed raw areas with fibrin, necrotic patches, and eschar on the dorsal aspect of the penis

The patient presented with superficial and deep second-degree thermal burns of the penis, extending from the base to the glans, with involvement of the scrotum. Total body surface area affected was approximately 10%, including the genital region and anterior neck. The neck and genital areas showed areas of necrosis and fibrin. No other body areas were affected. Detailed images of the burns are provided in Figures 1, 2.

Initial management and Foley catheterization

Upon admission, an 18-Fr Foley catheter was placed. Ten days later, it was replaced with a 16-Fr Foley catheter, which was removed upon discharge. Interconsultation with the general surgery service was requested for definitive management.

Surgical description

**Figure 3:**
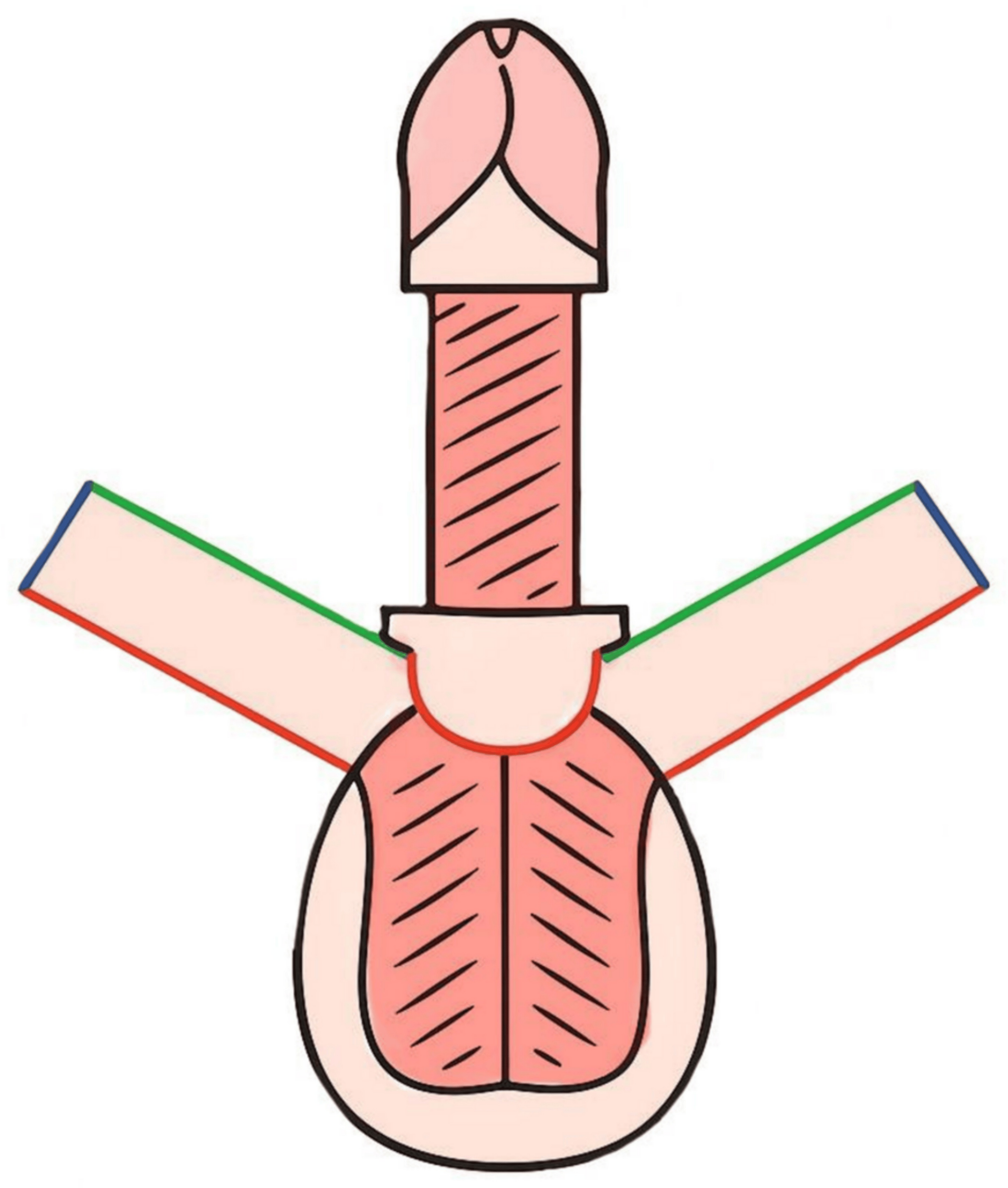
Inferior surgical view with flap harvest and rotation, demonstrating scrotal flap design and rotation A longitudinal midline incision is split into two mirrored flaps, each color-coded (red, blue, green) according to their final suture alignment Image credit: This is an original illustration created by the author Edgar Andrés Perea Celestín

**Figure 4:**
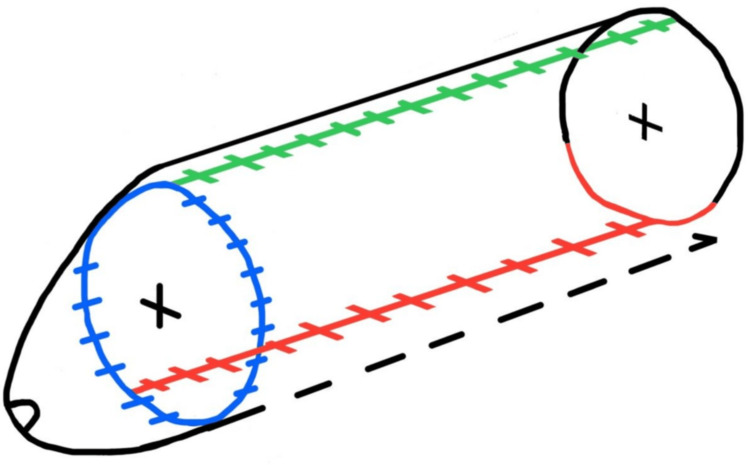
Three-dimensional design of flap reconstruction Preoperative surgical plan illustrating placement of flaps and expected suture lines using color coding: green (dorsal penile midline suture), blue (circumferential glans-shaft junction), and red (ventral midline penile suture) Image credit: This is an original illustration created by the author Edgar Andrés Perea Celestín

**Figure 5:**
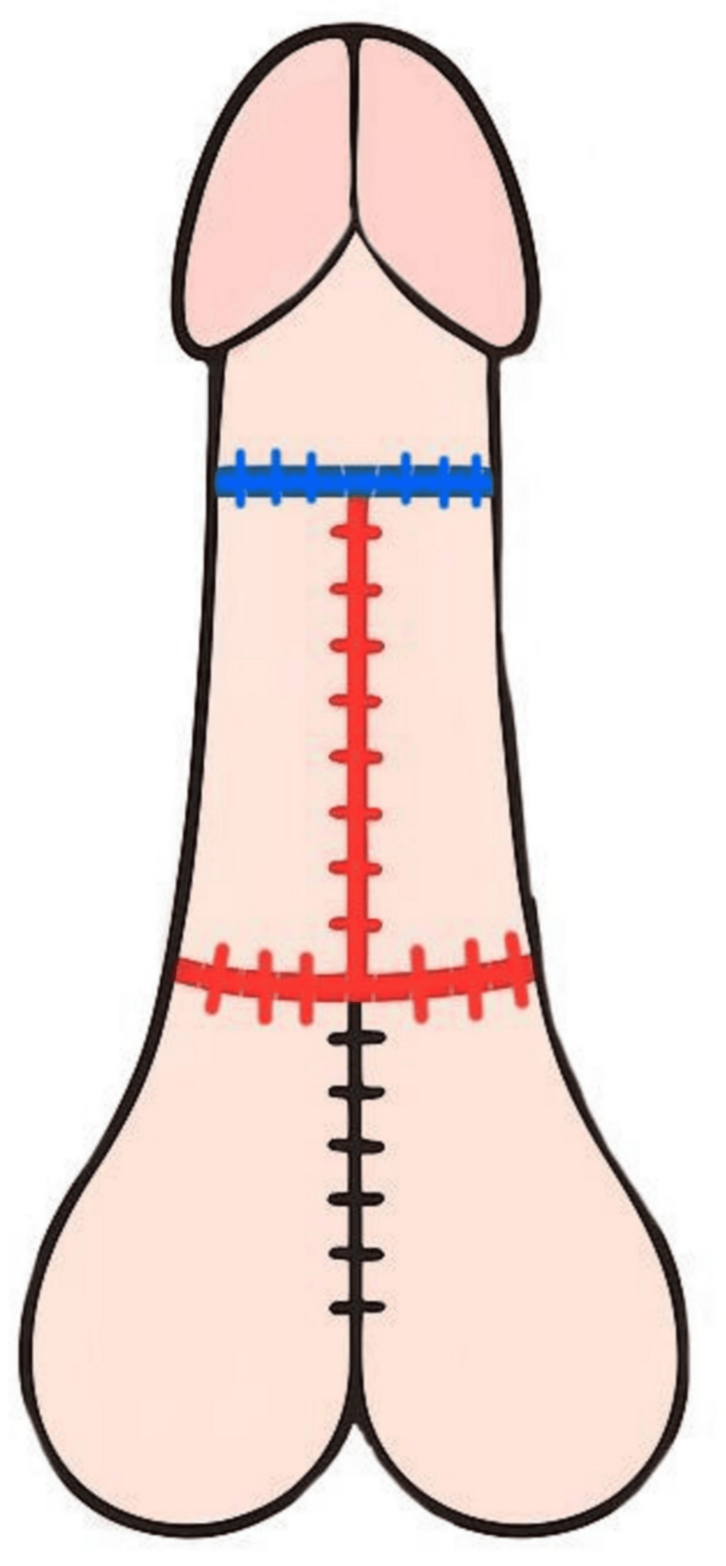
Inferior view of the penis and scrotum with suture placement after surgery Color-coded schematic showing postoperative suture locations: blue (circumferential suture at glans-shaft junction), red (midline ventral penile suture), and black (midline scrotal suture) Image credit: This is an original illustration created by the author Edgar Andrés Perea Celestín

**Figure 6:**
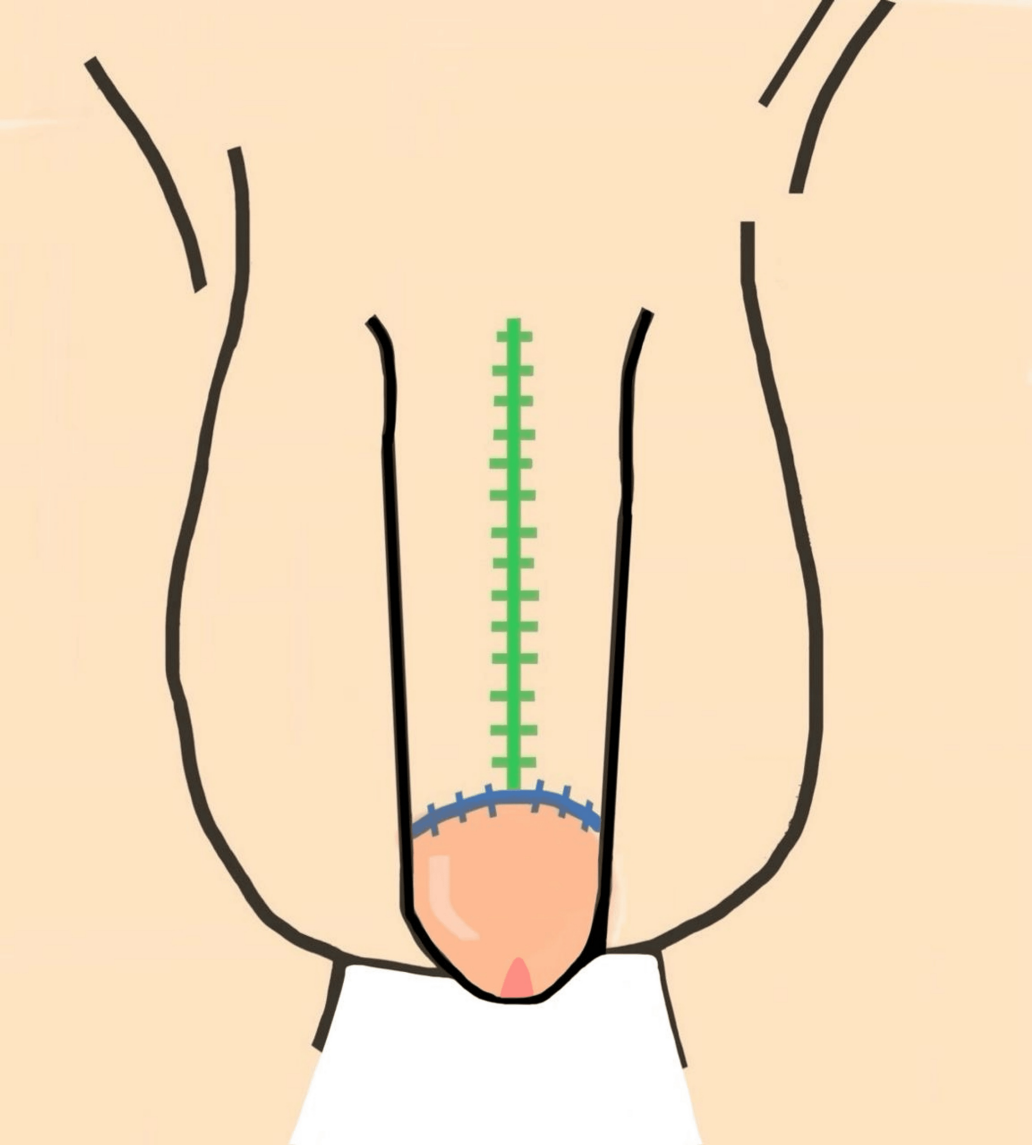
Frontal anatomical view of postoperative penis The dorsal penile suture (green) following flap rotation and alignment Image credit: This is an original illustration created by the author Edgar Andrés Perea Celestín

**Figure 7:**
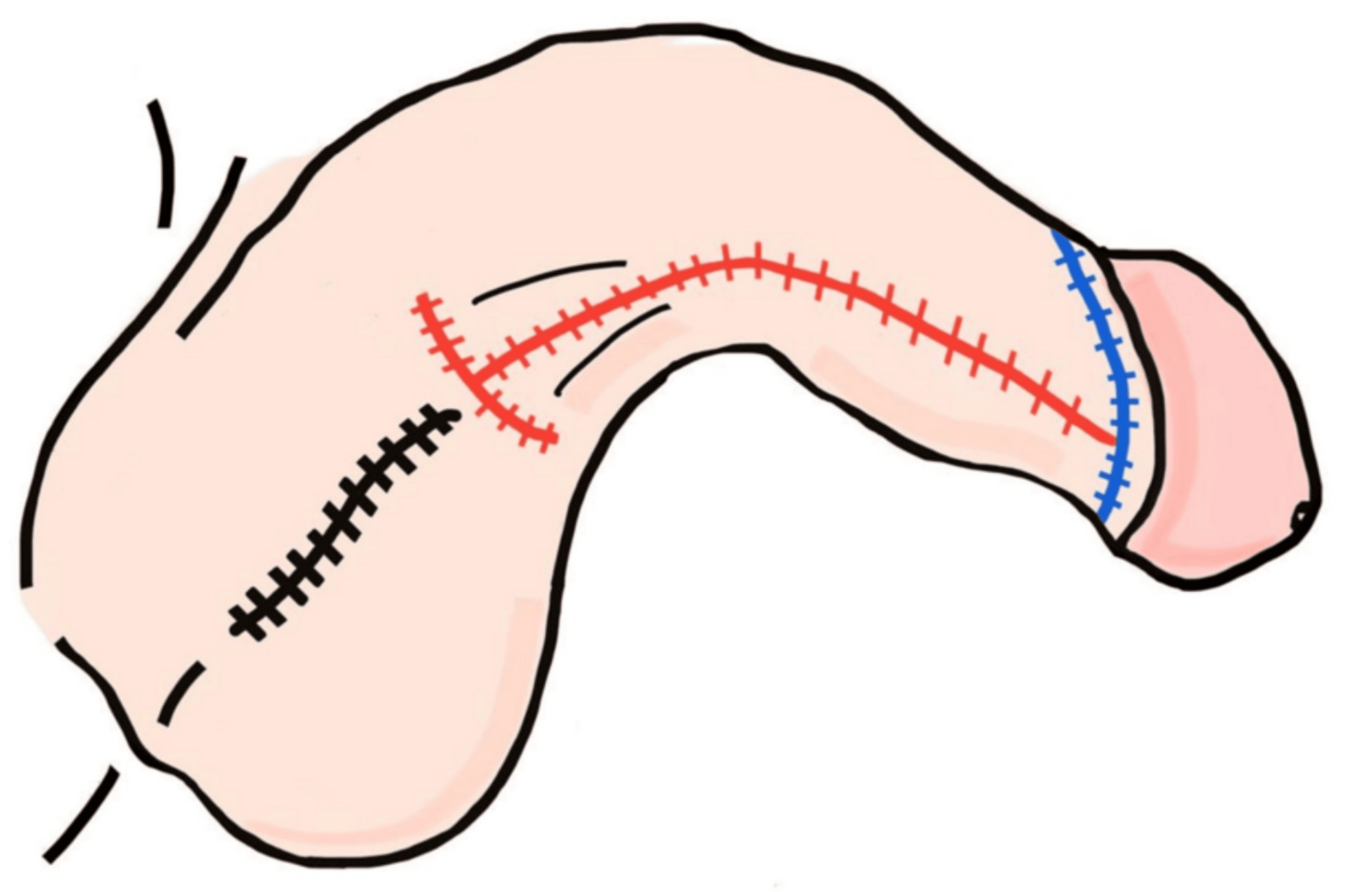
Oblique-lateral view of penis and scrotum after reconstruction Final suture positioning: black (scrotal midline), red (ventral penile midline), and blue (glans-shaft junction) Image credit: This is an original illustration created by the author Edgar Andrés Perea Celestín

**Figure 8:**
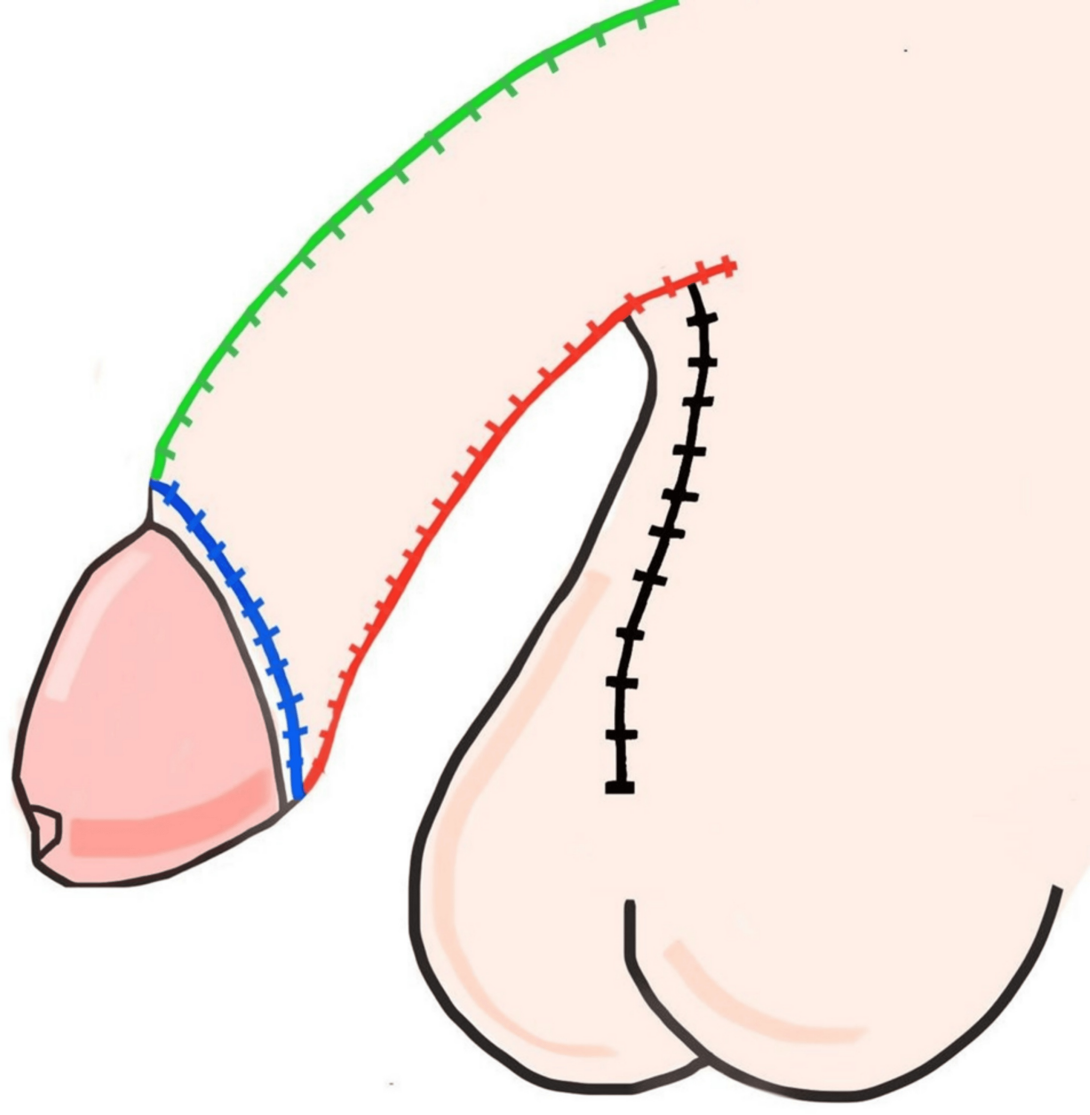
Lateral anatomical view of penis and scrotum Postoperative illustration showing lateral alignment of all three suture lines (green, red, blue) in anatomical position Image credit: This is an original illustration created by the author Edgar Andrés Perea Celestín

Under aseptic conditions, a 16-Fr Foley catheter was inserted to splint the urethra, and a cystostomy with an 18-Fr Foley catheter was performed for urinary diversion. The penile shaft was degloved, and a vertical fasciocutaneous scrotal flap was designed along the longitudinal axis of the scrotum with a random-pattern blood supply (approximately 12 cm in length). The flap was then rotated and sutured to cover the dorsal aspect of the penis, as illustrated in Figure 3. Anastomosis between the penile fascia and random dorsal penile vessels was performed to enhance flap perfusion and prevent venous congestion. The skin was closed, as shown in Figures 4-8.

Discharge and clinical outcomes

**Figure 9:**
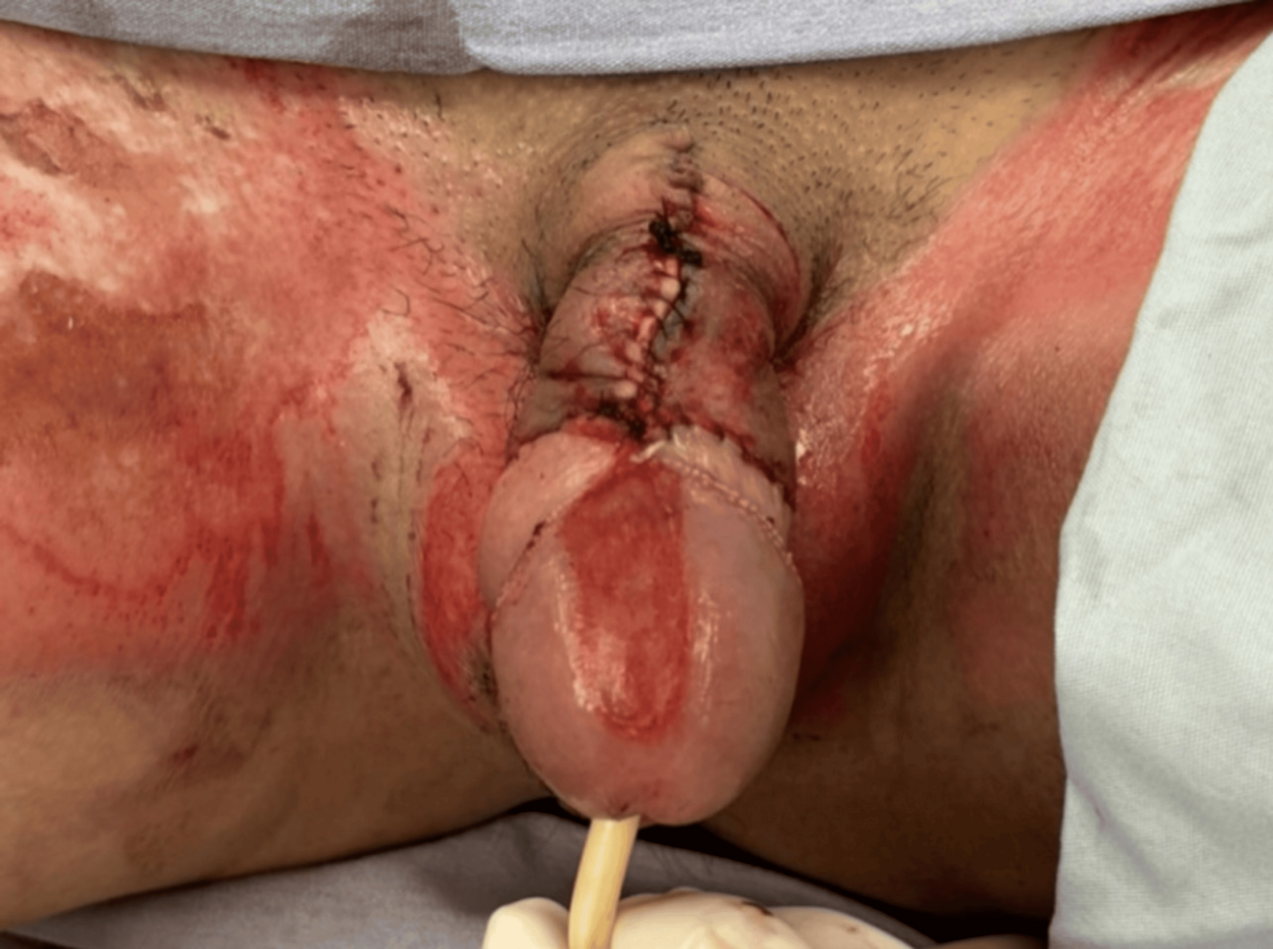
Immediate postoperative frontal view Clinical image showing the circumferential suture (blue), dorsal suture (green), and urethral stent in place with Foley catheter

**Figure 10:**
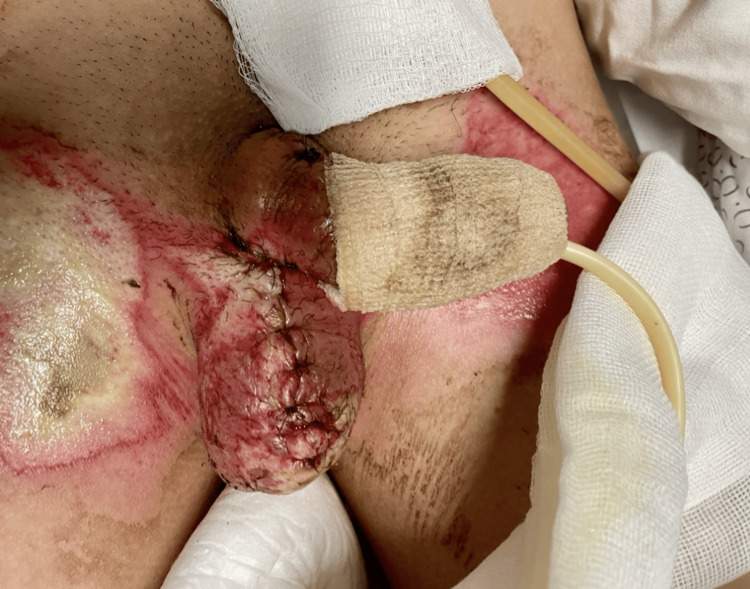
Scrotal view immediately after surgery Midline scrotal closure (black suture line) following rotation and flap reconstruction

**Figure 11:**
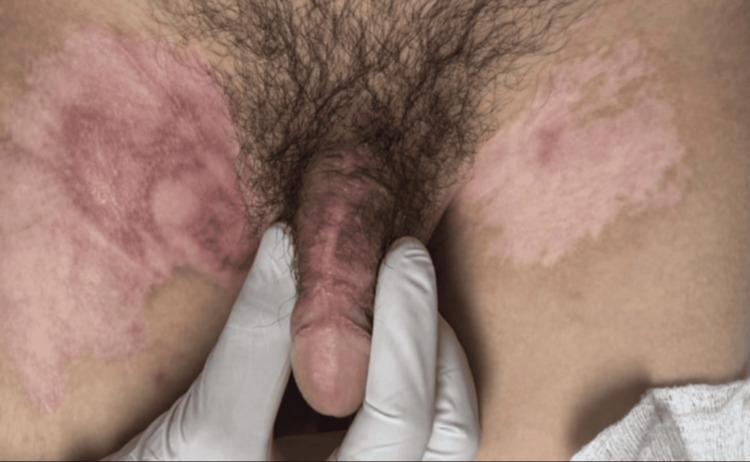
Dorsal penile view weeks after surgery Postoperative follow-up image showing excellent healing and esthetically acceptable scar formation on the dorsal penile shaft

**Figure 12:**
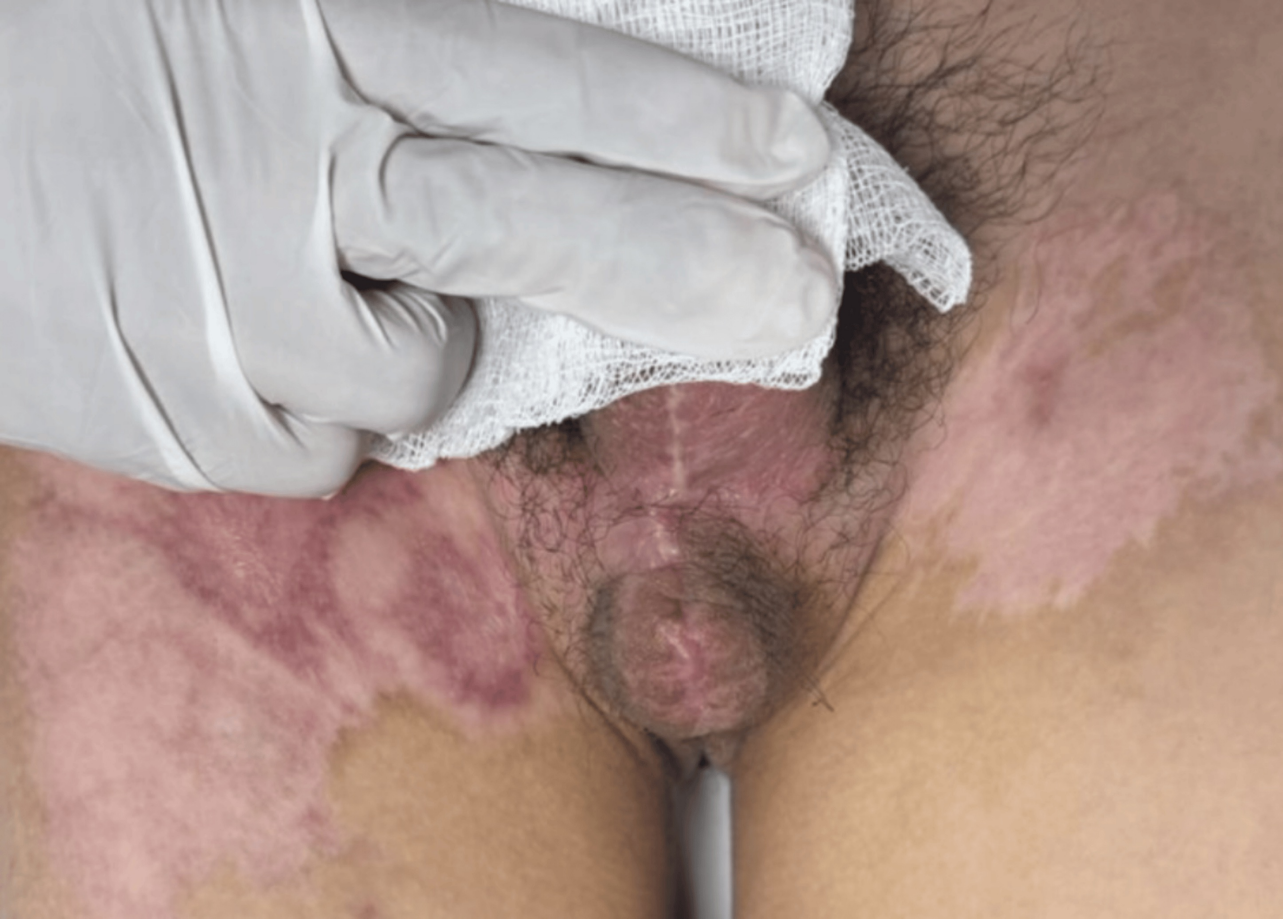
Scrotal view weeks after surgery Linear midline scrotal scar with good tissue integration and minimal contracture

Anatomical restoration of the genital area was achieved, with preserved tactile sensitivity and genital anatomy. Postoperative follow-up over two months in outpatient consultations showed complete healing without tissue contracture or retraction. The patient reported spontaneous voiding in an upright position and normal erectile function, with no hematuria or damage to the corpora cavernosa. No intraoperative or postoperative complications occurred, and the clinical course was favorable. The patient's maintained urinary and sexual function contributed positively to his mental and psychosocial health, reflecting the success of the surgical intervention. Immediate postoperative results are illustrated in Figures 9, 10. Figures 11, 12 show the outcomes during subsequent follow-up visits.

## Discussion

Burn injuries involving the genitalia have an estimated incidence ranging from 2.7% to 12.5%, depending on the type and location of the burn, with reported mortality rates of up to 67% in the literature [5]. Indications for penile reconstruction include various traumatic events, among which high-voltage electrical burns can be the most severe. Other reasons for reconstruction include congenital malformations, oncologic conditions, and gender-affirming surgery [4].

As previously noted, high-voltage electrical burns are relatively rare but carry high rates of morbidity and mortality. They represent the second most common type of burn injury to the genitalia, after chemical and thermal burns [5]. Such injuries significantly impair patients' physical and psychological well-being. Approximately 53% of affected patients develop erectile dysfunction, and many experience a high incidence of infections due to urinary and fecal contamination [6,7].

The penis is composed of corpora cavernosa, corpus spongiosum, fascia layers, dorsal neurovascular structures, tunica albuginea, and skin. High-voltage burns may necessitate extensive surgical debridement of the affected areas, predisposing the patient to postoperative complications [2]. Electrical burns produce tissue injury mainly by converting electrical energy into thermal energy, with damage influenced by tissue resistance and the path of current through the body [3,6]. Low-resistance tissues (nerves, vessels, mucosa, muscle) are more susceptible, while skin, bone, fat, and tendon show higher resistance, affecting injury severity. Prompt treatment is crucial to prevent severe contractures, necrosis, and infection.

The diagnostic evaluation and therapeutic management of patients with genital burns should include a thorough physical examination, laboratory testing, tetanus prophylaxis, and intravenous antibiotic therapy [8]. When indicated, bladder and rectal assessment via cystoscopy and sigmoidoscopy should be performed, with consideration for possible osseous injury [8]. Additional investigations, such as urinalysis, can help detect renal injury, as evidenced by myoglobinuria or hemoglobinuria [8]. Intensive hydration should be initiated to alkalinize urine, along with continuous cardiac monitoring, and surgical intervention should be performed when indicated [8].

Electrical burns can result from direct or indirect mechanisms, with tissue injury occurring through the conversion of electrical energy into thermal energy [6]. The severity of injury is inversely proportional to the resistance of the affected tissue and directly proportional to the voltage. Due to its high water content, the human body is an excellent electrical conductor; nerves, blood vessels, mucous membranes, and muscles are low-resistance tissues, while skin offers intermediate resistance, and bone, fat, and tendons exhibit relatively high resistance. Each tissue's resistance level determines the extent of damage sustained [6].

If not treated promptly, these injuries can progress to severe contractures, especially at the exit point [3]. Necrotic tissue (eschar) may develop, with a risk of bacterial invasion into adjacent tissues. The host immune response involves the release of cytokines, tumor necrosis factor, and interleukins [2]. Management extends beyond surgical treatment, as all patients require psychological rehabilitation, often including partner counseling [1]. The psychological impact is influenced by the degree of amputation, which can also increase difficulty with micturition and sexual intercourse [1]. Treatment should, therefore, be individualized and comprehensive.

The primary goals of surgical reconstruction are to preserve as much viable tissue as possible and achieve a tension-free repair that provides adequate penile length and girth for full, functional erections. It is critical to maintain voluntary micturition and the ability to void in a standing position, as well as to preserve protective sensation to prevent chronic skin degeneration. Additional objectives include preventing scar contracture, preserving the integrity of the corpora cavernosa, and achieving an acceptable cosmetic outcome that supports both physical and psychological well-being [2].

Reconstructive options include local pedicled flaps and free flaps (radial forearm, latissimus dorsi, anterolateral thigh, osteocutaneous fibula) [1]. Inguinal flaps offer certain advantages, including proximity to the recipient site, reliable vascular supply, and satisfactory sensory outcomes. However, the literature notes suboptimal esthetic results due to bulkiness at the reconstructed site and functional limitations from poor donor-site healing [1].

Conversely, the radial forearm free flap is widely used for its excellent aesthetic outcomes. However, it carries a relative disadvantage with complications such as fistula and urethral stricture, with reported incidence rates of up to 41% [4]. Urethral strictures typically occur at the urethral anastomosis site and can often be managed in an outpatient setting with periodic urethral dilations [4].

Scrotal flaps offer the possibility of reconstruction with donor tissue of similar characteristics, thereby reducing tension at the repair site, minimizing donor-site morbidity, and avoiding prolonged hospitalization [2]. The scrotal skin flap provides several advantages over other flap types, including favorable texture, reduced contracture tendency, and greater elasticity during erection. However, the literature on its use for coverage of extensive skin defects remains scarce [2].

Local tissue reconstruction offers multiple benefits, such as lower therapeutic failure rates, shorter operative time, reduced technical complexity, easier concealment of the donor-site scar, and potentially faster recovery [1]. The scrotal skin flap is a viable option for complete penile skin coverage, enabling maintenance of urethral patency and promoting adequate vascularization [9]. Preserving as much tissue as possible and ensuring an abundant blood supply to the injured areas are essential for optimal healing and reduced risk of necrosis. Nevertheless, as previously mentioned, there is limited published evidence on this technique.

## Conclusions

Modern reconstructive techniques have advanced to the point where the creation of a neophallus and neoscrotum with a visually natural appearance is achievable, often providing patients the ability to urinate while standing and to experience satisfactory sexual function. This case highlights the rarity and complexity of phalloplasty using scrotal flaps. Among the multiple approaches described, the use of scrotal flaps has gained attention due to their anatomical proximity, reliable vascularization, and skin characteristics that offer favorable texture and pigmentation, which contribute to satisfactory cosmetic and functional outcomes. In this patient, the scrotal flap resulted in successful urinary and sexual function, demonstrating its feasibility and effectiveness. Although the current literature on scrotal flap phalloplasty is limited, the reports available describe encouraging results with regard to both urinary function and patient sexual satisfaction. Nevertheless, it must be highlighted that there are no universally accepted guidelines defining a single gold-standard technique for phalloplasty, and surgical planning should therefore remain highly individualized, taking into account the patient's anatomy, clinical context, and personal expectations. Larger studies with long-term follow-up are needed to confirm outcomes, refine techniques, and establish evidence-based guidelines for this rare but important reconstructive procedure. Optimal outcomes are best achieved through multidisciplinary collaboration between plastic surgeons and urologists, while ongoing refinements and long-term follow-up studies will be essential to validate the promising results observed to date and to establish evidence-based recommendations for the future.
